# Radiological Society of North America (RSNA) 3D Printing Special Interest Group (SIG) clinical situations for which 3D printing is considered an appropriate representation or extension of data contained in a medical imaging examination: breast conditions

**DOI:** 10.1186/s41205-023-00171-1

**Published:** 2023-03-23

**Authors:** Elsa M. Arribas, Tatiana Kelil, Lumarie Santiago, Arafat Ali, Seetharam C. Chadalavada, Leonid Chepelev, Anish Ghodadra, Ciprian N. Ionita, Joonhyuk Lee, Prashanth Ravi, Justin R. Ryan, Adnan M. Sheikh, Frank J. Rybicki, David H. Ballard, RSNA Special Interest 3D Printing Breast Conditions Voting Group

**Affiliations:** 1 Division of Diagnostic Imaging, Department of Breast Imaging, University of Texas MD Anderson Cancer Center, Houston, TX 77030 USA; 2 Department of Radiology, University of California, 1600 Divisadero St, C250, Box 1667, San Francisco, CA 94115 USA; 3 Diagnostic Radiology, Henry Ford Medical Group, Henry Ford Hospital, 2799 W Grand Blvd, Detroit, MI 48202 USA; 4 Department of Radiology, University of Cincinnati College of Medicine, Cincinnati, OH USA; 5 Joint Department of Medical Imaging, University of Toronto, Toronto, ON Canada; 6 UPMC Department of Radiology, University of Pittsburgh School of Medicine, Pittsburgh, PA 15213 USA; 7 Department of Biomedical Engineering, Jacobs School of Medicine and Biomedical Sciences at the University at Buffalo, University at Buffalo School of Engineering and Applied Sciences, 8052 Clinical Translational Research Center, 875 Ellicott Street, Buffalo, NY 14203 USA; 8 University of Cincinnati College of Medicine, Cincinnati, OH 45267 USA; 9 3D Innovations Lab, Rady Children’s Hospital, San Diego, CA USA; 10 Department of Medical Imaging, Ottawa Hospital Research Institute (OHRI), The Ottawa Hospital, University of Ottawa, 501 Smyth Road, Ottawa, K1H 8L6 Canada; 11 Mallinckrodt Institute of Radiology, Washington University, St Louis, MO USA

## Abstract

The use of medical 3D printing has expanded dramatically for breast diseases. A writing group composed of the Radiological Society of North America (RSNA) Special Interest Group on 3D Printing (SIG) provides updated appropriateness criteria for breast 3D printing in various clinical scenarios. Evidence-based appropriateness criteria are provided for the following clinical scenarios: benign breast lesions and high-risk breast lesions, breast cancer, breast reconstruction, and breast radiation (treatment planning and radiation delivery).

**Supplementary Information:**

The online version contains supplementary material available at 10.1186/s41205-023-00171-1.

## Introduction

Currently, medical 3D printing is performed for a variety of indications. In 2018, the RSNA 3D printing SIG published appropriateness criteria for medical 3D printing for various clinical scenarios [1]. These included indications for breast 3D printing, which will now be revised and updated. The purpose of this document is to identify, vet, vote and publish a revised appropriate use criteria (AUC) for 3D printing in breast.

## Methods

The RSNA Special Interest Group (SIG) on 3D Printing Guidelines Committee has initiated and revised several documents regarding the Clinical scenarios for which 3D Printing is considered an appropriate representation or extension of data contained in a medical imaging examination. This document highlights appropriateness of medical 3D printing for clinical utilization, research, scientific and informational purposes within breast diseases. This work is loosely modeled after the American College of Radiology (ACR) Appropriateness Criteria® [2] in that the guidelines committee uses an evidence-based approach at scoring. Consensus among members is used when there is a paucity of evidence. Strength of evidence is determined by literature review.

The SIG Guidelines Chairperson oversees the ratings via a vote among Special Interest Group members at in-person meetings. The results of the ratings follow the following 1–9 format (with 9 being the most appropriate):

- 1–3, rarely appropriate. There is a lack of a clear benefit or experience that shows an advantage over usual practice.
- 4–6, maybe appropriate. There may be times when there is an advantage, but the data is lacking, or the benefits have not been fully defined.
- 7–9, usually appropriate. Data and experience show an advantage to 3D printing as a method to represent and/or extend the value of data contained in the medical imaging examination.

Clinical scenarios included in this document are stratified by histopathologic diagnosis and treatment. These include benign breast lesions and high-risk breast lesions, malignant breast lesions, breast reconstruction, and breast radiation (treatment planning and radiation delivery).

An exhaustive English language PubMed literature search was performed (May 2022) which enabled the querying and retrieval of pertinent clinical documents regarding the appropriateness of 3D printing in each of the scenarios. The supporting evidence was obtained through structured searches, as detailed in each category. For each category, from the pool of total results, the number of publications considered “relevant results” was curated by consensus between physicians with expertise in 3D printing and breast care. Relevant publications that were not retrieved by the structured PubMed search were manually entered. The following categories were excluded because they were considered outside the project scope (virtual & augmented reality, bioprinting, molecular biology, anthropomorphic models, and phantoms). All final components of this section were vetted and approved by vote of Special Interest Group members virtually at the July 20^th^, 2022 SIG Appropriateness Committee Meeting. Afterwards, 2-week period for comments by SIG members was posted on the SIG’s members-only online forum. All included studies were graded with a strength of evidence assessment according to ACR Appropriateness Criteria Evidence Document.

## Results

1. **Benign breast lesions and high-risk breast lesions:** Benign breast diseases are common and include a wide range of entities [3]. The most common of these entities, fibrocystic change, is clinically observed in up to 50% of women and found histologically in 90% of women [3]. Fibroadenomas are the next most common benign breast disease occurring in 15–23% of women [4]. Benign breast lesions include fibrocystic change, benign breast masses, inflammatory, and peripartum conditions [3, 4].
  High-risk breast lesions may confer an increased risk for breast cancer, may be associated with a higher risk for future breast cancer, and may be precursors in breast carcinogenesis. Management of high-risk lesions after core needle biopsy may include close imaging and clinical follow-up or excisional biopsy to evaluate for cancer [5–8]. High-risk lesions include flat epithelial atypia, atypical ductal hyperplasia, lobular neoplasia, radial scar, papillary lesions, and mucocele-like lesions [9].
  Surgical management of these entities may be needed in cases where cosmesis is altered or when symptom relief is needed. Surgical management may impact developing breast tissue in young women leading to alterations in its proper development [10]. Therefore, careful understanding of the anatomy may minimize the deleterious effects of surgery in benign breast disease.
**PubMed Search:** ((3D printing) AND (fibrocystic change)) OR ((3D printing) AND (benign breast masses)) OR ((3D printing) AND (mastitis)) OR ((3D printing) AND (galactocele)) OR ((rapid prototyping) AND (fibrocystic change)) OR ((rapid prototyping) AND (benign breast masses)) OR ((rapid prototyping) AND (mastitis) OR ((rapid prototyping) AND (galactocele)). ((3D printing) AND (flat epithelial atypia)) OR ((3D printing) AND (atypical ductal hyperplasia)) OR ((3D printing) AND (lobular neoplasia)) OR ((3D printing) AND (radial scar)) OR ((3D printing) AND (papillary lesions) OR ((3D printing) AND (mucocele-like lesions)) OR ((rapid prototyping) AND (flat epithelial atypia)) OR ((rapid prototyping) AND (atypical ductal hyperplasia)) OR ((rapid prototyping) AND (lobular neoplasia) OR ((rapid prototyping) AND (radial scar)) OR ((rapid prototyping) AND (papillary lesions)) OR ((rapid prototyping) AND (mucocele-like lesions))
**PubMed Results:** No results found.
**Preliminary rating: 1**
2. **Malignant breast lesions:** Breast cancer is the most common solid malignancy in women in the United States [11]. Approximately 281,550 new breast cancers are estimated to be diagnosed in 2021 constituting 15% of all new cancers diagnosed in the United States. The overall lifetime risk of developing breast cancer for women in the United States is 12.9%. Advancements in diagnostic tests and treatments have led to decreasing death rates of 1.4% per year from 2009 to 2018 [11, 12]. Breast malignancies include ductal carcinoma in situ ductal (DCIS) and invasive breast carcinomas [13]. Understanding the extent of disease at the time of diagnosis allows appropriate staging and determination of prognosis and survival in addition to selection of suitable surgical options [14]. They are also an effective tool to reduce decision conflict in patients and enhance the informed consent [15]. 3D printed models have the ability of depicting the extent of disease and relationships of sensitive anatomy [16]. This information can be translated into 3D printed surgical guides that may accurately localize cancers and achieve negative surgical margins [17–19]. Additional possible outcomes that need to be studied/determined: reducing operating time, enhancing utilization of new oncoplastic techniques, and improving patient outcomes.
**PubMed Search:** ((3D printing) AND (breast cancer) OR ((rapid prototyping) AND (breast cancer))
**PubMed Results: 14 **[15, 16, 18–29]
  a Schulz-Wendtland R, Harz M, Meier-Meitinger M, et al. Semi-automated delineation of breast cancer tumors and subsequent materialization using three-dimensional printing (rapid prototyping). J Surg Oncol. 2017;115(3):238–242.
  b Santiago L, Volk RJ, Checka CM, et al. Acceptability of 3D‐printed breast models and their impact on the decisional conflict of breast cancer patients: A feasibility study. Journal of surgical oncology. 2021;123(5):1206–1214.
  c Barth RJ, Krishnaswamy V, Paulsen KD, et al. A Patient-Specific 3D-Printed Form Accurately Transfers Supine MRI-Derived Tumor Localization Information to Guide Breast-Conserving Surgery. Annals of Surgical Oncology. 2017;24(10):2950–2956.
  d Rao N, Chen K, Yang Q, Niu J. Proof-of-Concept Study of 3-D-Printed Mold-Guided Breast-Conserving Surgery in Breast Cancer Patients. Clin Breast Cancer. 2018;18(5):e769-e772.
  e Ko BS, Kim N, Lee JW, et al. MRI-based 3D-printed surgical guides for breast cancer patients who received neoadjuvant chemotherapy. Scientific Reports. 2019;9(1):11,991.
  f Fernandez RAS, Lau RWH, Yu PSY, Siu ICH, Chan JWY, Ng CSH. Use of custom made 3-dimensional printed surgical guide for manubrio-sternal resection of solitary breast cancer metastasis: case report. AME Case Rep. 2020;4:12.
  g Wu ZY, Alzuhair A, Kim H, et al. Magnetic resonance imaging based 3-dimensional printed breast surgical guide for breast-conserving surgery in ductal carcinoma in situ: a clinical trial. Sci Rep. 2020;10(1):18,534.
  h Ock J, Lee S, Kim T, et al. Accuracy evaluation of a 3D printing surgical guide for breast-conserving surgery using a realistic breast phantom. Computers in Biology and Medicine. 2021;137:104,784.
  i Wu ZY, Kim HJ, Lee J, et al. Breast-conserving surgery with 3D-printed surgical guide: a single-center, prospective clinical study. Sci Rep. 2021;11(1):2252.
  j Wu ZY, Kim GB, Choi S, Lee S, Kim N, Ko B. Breast-Conserving Surgery after Neoadjuvant Chemotherapy Using a Three-Dimensional-Printed Surgical Guide Based on Supine Magnetic Resonance Imaging: A Case Report. J Breast Cancer. 2021;24(2):235–240.
  k Wu ZY, Lee YJ, Shin Y, et al. Usefulness of 3-Dimensional-Printed Breast Surgical Guides for Undetectable Ductal Carcinoma In Situ on Ultrasonography: A Report of 2 Cases. J Breast Cancer. 2021;24(3):349–355.
  l Wu ZY, Kim GB, Lee S, Choi SH, Kim N, Ko B. Case Report: A 3D-Printed Surgical Guide for Breast-Conserving Surgery After Neoadjuvant Chemotherapy. Front Oncol. 2021;11:633,302.
  m Lee HS, Kim HJ, Chung IY, et al. Usefulness of 3D-surgical guides in breast conserving surgery after neoadjuvant treatment. Sci Rep. 2021;11(1):3376.
  n Santiago L, Adrada BE, Caudle AS, Clemens MW, Black DM, Arribas EM. The role of three‐dimensional printing in the surgical management of breast cancer. Journal of surgical oncology. 2019;120(6):897–902.
**Preliminary Rating: 7**
3. **Breast reconstruction:** Breast reconstruction surgeries include either implant-based or autologous flap reconstructions. In autologous flap reconstructions, 3D printed models have been shown to facilitate the intramuscular dissection of perforator vessels by depicting the course and trajectory of the subfascial vascular tree and allowing the surgeon to view the model from various vantage points [24, 30, 31]. Improved understanding of the course of perforators and perfusion characteristics may be useful in reducing the risk of fat necrosis, unintended vessel injury, and the need for secondary procedures [32]. 3D models can be used for accurate analysis of breast volume, shape, and contour preoperatively and to facilitate the shaping and positioning of the flap intraoperatively, leading to symmetric surgical outcomes [33, 34].
**PubMed Search:** ((3D printing) AND (breast reconstruction) OR ((rapid prototyping) AND (breast reconstruction)
**PubMed Results: 11** [30–40]
  a Jablonka EM, Wu RT, Mittermiller PA, Gifford K, Momeni A. 3-DIEPrinting: 3D-printed models to assist the intramuscular dissection in abdominally based microsurgical breast reconstruction. Plastic and Reconstructive Surgery Global Open. 2019;7(4).
  b Chae MP, Rozen WM, McMenamin PG, Findlay MW, Spychal RT, Hunter-Smith DJ. Emerging applications of bedside 3D printing in plastic surgery. Frontiers in surgery. 2015;2:25.
  c Chae MP, Hunter-Smith DJ, Rostek M, Smith JA, Rozen WM. Enhanced preoperative deep inferior epigastric artery perforator flap planning with a 3D-printed perforasome template: technique and case report. Plastic and Reconstructive Surgery Global Open. 2018;6(1).
  d Chae MP, Hunter-Smith DJ, Spychal RT, Rozen WM. 3D volumetric analysis for planning breast reconstructive surgery. Breast Cancer Res Treat. 2014;146(2):457–460.
  e Hummelink S, Verhulst AC, Maal TJ, Ulrich DJ. Applications and limitations of using patient-specific 3D printed molds in autologous breast reconstruction. European journal of plastic surgery. 2018;41(5):571–576.
  f DeFazio MV, Arribas EM, Ahmad FI, et al. Application of Three-Dimensional Printed Vascular Modeling as a Perioperative Guide to Perforator Mapping and Pedicle Dissection during Abdominal Flap Harvest for Breast Reconstruction. J Reconstr Microsurg. 2020;36(5):325–338.
  g Mehta S, Byrne N, Karunanithy N, Farhadi J. 3D printing provides unrivalled bespoke teaching tools for autologous free flap breast reconstruction. Journal of Plastic, Reconstructive & Aesthetic Surgery. 2016;69(4):578–580.
  h Chae MP, Hunter-Smith DJ, Chung RD, Smith JA, Rozen WM. 3D-printed, patient-specific DIEP flap templates for preoperative planning in breast reconstruction: a prospective case series. Gland Surg. 2021;10(7):2192–2199.
  i Chen K, Feng CJ, Ma H, et al. Preoperative breast volume evaluation of one-stage immediate breast reconstruction using three-dimensional surface imaging and a printed mold. J Chin Med Assoc. 2019;82(9):732–739.
  j Ogunleye AA, Deptula PL, Inchauste SM, et al. The utility of three-dimensional models in complex microsurgical reconstruction. Arch Plast Surg. 2020;47(5):428–434.
  k Tomita K, Yano K, Taminato M, Nomori M, Hosokawa K. DIEP Flap Breast Reconstruction in Patients with Breast Ptosis: 2-Stage Reconstruction Using 3-Dimensional Surface Imaging and a Printed Mold. Plast Reconstr Surg Glob Open. 2017;5(10):e1511.
**Preliminary rating: 8**
4. **Breast Radiation (Treatment planning and Radiation delivery):** 3D printing can be used to design customized patient specific boluses and shields that allow homogeneous distribution of radiation dose to the area of interest while sparing adjacent normal tissue [41]. 3D printing can also be used to create customized brachytherapy templates where a radiation source is implanted next to the area requiring treatment. 3D printed customized brachytherapy templates provide a better fit for patients and are less prone to shift due to movement. [42]
**PubMed Search:** ((3D printing) AND (breast radiation) OR ((rapid prototyping) AND (breast radiation)
**PubMed Results: 8** [17, 42–48]
  a Poulin E, Gardi L, Fenster A, Pouliot J, Beaulieu L. Towards real-time 3D ultrasound planning and personalized 3D printing for breast HDR brachytherapy treatment. Radiother Oncol. 2015;114(3):335–338.
  b Aristei C, Lancellotta V, Piergentini M, et al. Individualized 3D-printed templates for high-dose-rate interstitial multicathether brachytherapy in patients with breast cancer. Brachytherapy. 2019;18(1):57–62.
  c Yang K, Park W, Ju SG, et al. Heart-sparing radiotherapy with three-dimensional printing technology after mastectomy for patients with left breast cancer. Breast J. 2019;25(4):682–686.
  d He C, Zhang S, Shi L. Three-Dimensionally-Precise Breast Conformal Device for IMRT in Breast Cancer Patients Treated With Breast-Conserving Surgery-A Pilot Randomized Controlled Trial. Technol Cancer Res Treat. 2020;19:1,533,033,820,971,563.
  e Robar JL, Moran K, Allan J, et al. Intrapatient study comparing 3D printed bolus versus standard vinyl gel sheet bolus for postmastectomy chest wall radiation therapy. Pract Radiat Oncol. 2018;8(4):221–229.
  f Park SY, Choi CH, Park JM, Chun M, Han JH, Kim JI. A Patient-Specific Polylactic Acid Bolus Made by a 3D Printer for Breast Cancer Radiation Therapy. PLoS One. 2016;11(12):e0168063.
  g Poulin E, Gardi L, Fenster A, Pouliot J, Beaulieu L. A novel approach for real-time, personalized breast HDR brachytherapy treatment using 3D printing technology. Brachytherapy. 2014;13:S18.
  h Park K, Park S, Jeon MJ, et al. Clinical application of 3D-printed-step-bolus in post-total-mastectomy electron conformal therapy. Oncotarget. 2017;8(15):25,660–25,668.
**Preliminary rating: 6**

Table 1 provides a summary of evidence based guidelines to define and support the use of 3D printing for patients with breast conditions. The citations included in forming the appropriateness criteria have been detailed above and the strength of evidence assessment is included in Additional file 1.

**Table 1:** Appropriateness Ratings for Breast Diseases Indications

Clinical Condition	Rating	Rating driven by	References	Study Quality (Additional file 1)	Number of patients combined	Number of patients in the largest series (reference)
Benign breast lesions & High-risk breast lesions	1	LA	0	NA	NA	NA
Malignant breast lesions	7	B	15, 16, 18–29	C2: 2 C3: 5 C4: 7	207	88
Breast reconstruction	8	B	30–40	C2: 3 C3: 2 C4: 6	222	116
Breast radiation (treatment planning and radiation delivery)	7	B	17, 42–48	C1: 1 C2: 3 C3: 2 C4: 3	123	30

Literature appraisal = LA; Literature appraisal and Expert Opinion = B; Category = C; Not available = NA

The “Rating driven by” column denotes if the primary decision for the condition’s rating was decided primarily through discussion of the available literature (LA), expert opinion (EO) or both (B). The “Study quality” column reflects the graded strength of evidence assessment according to ACR Appropriateness Criteria Evidence Document [2] (individual ratings available in Additional file 1). The highest/most robust level of evidence is ‘Category 1’ and the lowest is ‘Category 4’

## Limitations

Limitations of this work include its lack of objective data collection and inferential statistics. Although such an analysis would be desirable, it is not practical with most published breast related applications due to the small number of publications and patients. PubMed search terms, were based on prior search terminology from previously published guidelines [1]. The RSNA 3D Printing SIG is comprised of physicians (primarily radiologists), imaging scientists, biomedical engineers, and other 3D printing experts, the voting group did not have direct input from surgeons, breast imagers, or collaboration from a breast surgical or breast oncology professional organization. Future iterations should aim for such collaboration.

## Conclusion

This document provides updated appropriateness criteria for 3D printing in breast conditions. Adoption of common clinical standards regarding appropriate use, information and material management, and quality control are needed to ensure the greatest possible clinical benefit from 3D printing. With accruing evidence for in 3D printing, this consensus guideline document, created by the members of the RSNA 3D printing Special Interest Group, will provide a reference for clinical standards of 3D printing. The document will be periodically refined, based on expanding clinical applications and growing medical literature.

## Supplementary Information

**Additional file 1.**

## Data Availability

The datasets used and/or analyzed during the current study are available from the corresponding author on reasonable request.
