# Supplementary material for: Radiological Society of North America (RSNA) 3D Printing Special Interest Group (SIG) clinical situations for which 3D printing is considered an appropriate representation or extension of data contained in a medical imaging examination: breast conditions
Source: 3D Print Med. 2023 Mar 23;9:8. doi: 10.1186/s41205-023-00171-1 (PMC10037829; doi:10.1186/s41205-023-00171-1)
Supplement: Supplementary file 1 — Additional file 1. [file 41205_2023_171_MOESM1_ESM.docx]

**Clinical Situations for which 3D Printing is Considered an Appropriate Representation or Extension of Data Contained in Medical Imaging Examination: Breast Conditions**

**RSNA Special Interest Group on 3D Printing**

**Additional file 1: Strength of Evidence**

| **Reference** | | **Study Type** | **Patients/**  **Events** | **Study Objective**  **(Purpose of Study)** | **Study Results** | **Study Quality** |
| --- | --- | --- | --- | --- | --- | --- |
| 15 | Santiago, L., et al., Acceptability of 3D‐printed breast models and their impact on the decisional conflict of breast cancer patients: A feasibility study. Journal of surgical oncology, 2021. 123(5): p. 1206-1214. | Observational Tx | 25 | To evaluate the acceptability and impact of 3D-printed breast models (3D-BMs) on treatment-related decisional conflict (DC) of breast cancer patients. | DC surveys before and after 3D-BM review and 3D-BM acceptability surveys were completed by 25 patients. Patients rated the acceptability of the 3D-BM as good/excellent in understanding their condition (24/24), understanding disease size (25/25), 3D-BM detail (22/25), understanding their surgical options (24/25), encouraging to ask questions (23/25), 3D-BM size (24/25), and impartial to surgical options (17/24). There was a significant reduction in the overall DC post-3D-BM review, indicating patients became more assured of their treatment choice (p = 0.002). Reduction post-3D-BM review was also observed in the uncertainty (p = 0.012), feeling informed about options (p = 0.005), clarity about values (p = 0.032), and effective (p = 0.002) Decisional Conflict Scale subscales. | 2 |
| 16 | Schulz-Wendtland, R., et al., Semi-automated delineation of breast cancer tumors and subsequent materialization using three-dimensional printing (rapid prototyping). J Surg Oncol, 2017. 115(3): p. 238-242.. | Review/Other Dx | 5 | To explore facilities for the semi-automated delineation of breast cancer tumors and to assess the feasibility of 3D printing of breast cancer tumors. | The tumor volumes calculated from the different 3D methods appeared to be comparable. Tumor models with volumes between 325 mm3 and 7,770 mm3 were printed and compared with the models rendered from MRI. The materialization of the tumors reflected the computer models of them. | 4 |
| 17 | He C, Zhang S, Shi L. Three-Dimensionally-Precise Breast Conformal Device for IMRT in Breast Cancer Patients Treated With Breast-Conserving Surgery-A Pilot Randomized Controlled Trial. Technol Cancer Res Treat. 2020;19:1533033820971563. | Experimental Tx | 30 | To examine the accuracy and efficiency of breast radiotherapy after breast-conserving surgery of a novel 3-dimensional (3D) printing tissue compensator technology, the 3D-precise breast conformer, compared with a usual compensator and an unstructured compensator. | The 3D-BCT showed the best homogeneity index (HI) (0.08 ± 0.03) and conformity index (CI) (0.95 ± 0.03), while the NST group showed the worst HI (0.34 ± 0.07) and CI (0.78 ± 0.06), with the ST group between the 2 (HI: 0.15 ± 0.05; CI: 0.87 ± 0.04) (all P < 0.01). The common tissue compensation membrane could lead to 95-100% of the prescription dose covering 85-95% of the target volume, and the uniformity and conformability of the target dose were improved overall compared with the NST group. In the 3D-BCT group, 100% of the prescription dose covered the target volume of 95-100%. | 2 |
| 18 | Ko BS, Kim N, Lee JW, et al. MRI-based 3D-printed surgical guides for breast cancer patients who received neoadjuvant chemotherapy. Scientific Reports. 2019;9(1):11991. | Experimental Tx | 5 | No current guidance device exists that allows for estimation of an accurate range for breast-conserving surgery after neoadjuvant chemotherapy. We therefore developed three-dimensional (3D) printed surgical guides for breast-conserving surgery in breast cancer patients after neoadjuvant chemotherapy and evaluated their clinical utility. To the best of our knowledge, this is the first study to apply a surgical guide to breast cancer patients. | Out of the five patients who participated in the study, all patients had clear resection margins, and two patients experienced complete pathological remission. There were no recurrences during the median follow-up period of 21.9 months. Thus, our newly-developed 3D-printed surgical guides were useful for accurately marking the extent of breast tumor based on pretreatment magnetic resonance images, which is important for designating the extent of surgery needed in patients who have received neoadjuvant chemotherapy. | 3 |
| 19 | Barth RJ, Krishnaswamy V, Paulsen KD, et al. A Patient-Specific 3D-Printed Form Accurately Transfers Supine MRI-Derived Tumor Localization Information to Guide Breast-Conserving Surgery. Annals of Surgical Oncology. 2017;24(10):2950-2956. | Experimental Tx | 19 | Patients underwent preoperative supine magnetic resonance imaging (MRI). A radiologist outlined the tumor edges on consecutive images, creating a three-dimensional (3D) view of its location. Using 3D printing, a bra-like plastic form (the Breast Cancer Locator [BCL]) was fabricated, with features that allowed a surgeon to (1) mark the edges of the tumor on the breast surface; (2) inject blue dye into the breast 1 cm from the tumor edges; and (3) place a wire in the tumor at the time of surgery. | Information on breast cancer location and shape derived from a supine MRI can be transferred safely and accurately to patients in the operating room using a 3D-printed form. | 3 |
| 20 | Fernandez RAS, Lau RWH, Yu PSY, Siu ICH, Chan JWY, Ng CSH. Use of custom made 3-dimensional printed surgical guide for manubrio-sternal resection of solitary breast cancer metastasis: case report. AME Case Rep. 2020;4:12. | Review/Other-Tx | 1 | We describe our unique approach of using 3-dimensional (3D)-computed tomography (CT) image segmentation planning for reconstructing desirable resection boundaries, design of ideal superficial and deep surgical resection guides, and followed by 3D printing of guides using autoclavable thermoplastic for use during surgery. | The surgical guides over the ribs and sternum rapidly and accurately define resection lines intraoperatively, achieve good surgical margins, and could reduce resection and reconstruction related morbidity for performing complex surgical resection of the chest wall. The patient was discharged 2 weeks postoperatively and remained free from local recurrence on CT scan 1-year after resection. | 4 |
| 21 | Rao N, Chen K, Yang Q, Niu J. Proof-of-Concept Study of 3-D-Printed Mold-Guided Breast-Conserving Surgery in Breast Cancer Patients. Clin Breast Cancer. 2018;18(5):e769-e772. | Experimental Tx | 8 | In this proof-of-concept study, we proposed 3-D-printed mold-guided breast-conserving surgery (BCS) in breast cancer patients. | We performed 3-D-printed mold-guided BCS in 8 breast cancer patients. All patients had negative surgical margins, confirmed by intraoperative and postoperative pathologic examinations. | 3 |
| 22 | Wu ZY, Alzuhair A, Kim H, et al. Magnetic resonance imaging based 3-dimensional printed breast surgical guide for breast-conserving surgery in ductal carcinoma in situ: a clinical trial. Sci Rep. 2020;10(1):18534. | Experimental Tx | 11 | A three-dimensional printed (3DP) breast surgical guide (BSG) was developed using information obtained from supine magnetic resonance imaging (MRI) and 3D printing technology and it was used for treating patients with breast cancer. Authors describe their experience with the application of the BSG for patients with DCIS. | Direct demarcation of the tumor extent in the breast and a pain-free procedure are the advantages of using 3DP-BSG in patients with DCIS. | 3 |
| 23 | Ock J, Lee S, Kim T, et al. Accuracy evaluation of a 3D printing surgical guide for breast-conserving surgery using a realistic breast phantom. Computers in Biology and Medicine. 2021;137:104784. | Review/Other-Tx | 1 | To prevent recurrence after breast-conserving surgery (BCS), it is imperative to secure a clear resection margin, and magnetic resonance imaging (MRI) is useful for predicting this. Although MRI is highly accurate in predicting the extent of a tumor, it is difficult to quantitatively mark the tumor area directly on the patient's breast skin using MRI. Therefore, we developed a 3D-printed breast surgical guide (3DP-BSG). | This 3DP-BSG exhibits high accuracy in tumor targeting and is expected to facilitate precise BCS by providing a quantitative measure of the tumor area to surgeons. | 4 |
| 24 | Wu ZY, Kim HJ, Lee J, et al. Breast-conserving surgery with 3D-printed surgical guide: a single-center, prospective clinical study. Sci Rep. 2021;11(1):2252. | Experimental Tx | 88 | To facilitate precise tumor resection at the time of breast-conserving surgery (BCS), we developed and implemented a magnetic resonance imaging (MRI)-based three-dimensional-printed (3DP) breast surgical guide (BSG). | In 93.3% of the cases, the resection margin was tumor-free in the permanent pathology. The mean pathological tumor size was 1.7 ± 1.0 cm, and the mean distance from the tumor to the border was 1.5 ± 1.0 cm. This exploratory study showed that the tumor area on the MRI could be directly displayed on the breast when using a 3DP BSG for BCS, thereby allowing precise surgery and safe tumor removal. | 3 |
| 25 | Wu ZY, Kim GB, Choi S, Lee S, Kim N, Ko B. Breast-Conserving Surgery after Neoadjuvant Chemotherapy Using a Three-Dimensional-Printed Surgical Guide Based on Supine Magnetic Resonance Imaging: A Case Report. J Breast Cancer. 2021;24(2):235-240. | Review/Other-Tx | 1 | To increase the accuracy of tumor resection, we used a 3-dimensional-printed breast surgical guide based on magnetic resonance imaging (MRI) in the supine position for a breast cancer patient who underwent breast-conserving surgery after NACT. | Using this device, the breast tumor with apparent therapeutic changes after NACT on imaging was successfully removed with clear resection margins by identifying the original tumor site in the affected breast. Irrespective of whether the residual tumor area after NACT is well defined, it is possible to confirm and target the tumor area on pre-NACT MRI using this device. | 4 |
| 26 | Wu ZY, Lee YJ, Shin Y, et al. Usefulness of 3-Dimensional-Printed Breast Surgical Guides for Undetectable Ductal Carcinoma In Situ on Ultrasonography: A Report of 2 Cases. J Breast Cancer. 2021;24(3):349-355. | Review/Other-Tx | 2 | Here, we report the application of MRI-based individualized 3-dimensional (3D)-printed breast surgical guides (BSGs) for patients with breast cancer. | We successfully resected indeterminate and suspicious lesions that were only detected using preoperative MRI, and the final histopathologic results confirmed DCIS with clear resection margins. MRI guidance combined with 3D-printed BSGs can be used for DCIS localization, especially for lesions easily detectable using MRI only. | 4 |
| 27 | Wu ZY, Kim GB, Lee S, Choi SH, Kim N, Ko B. Case Report: A 3D-Printed Surgical Guide for Breast-Conserving Surgery After Neoadjuvant Chemotherapy. Front Oncol. 2021;11:633302. | Review/Other-Tx | 1 | For breast-conserving surgery, the development of a guidance device to accurately estimate the resection area is imperative. | We produced a three-dimensional (3D)–printed breast surgical guide (BSG) based on prone and supine magnetic resonance imaging (MRI). This device was tested on a patient who underwent breast-conserving surgery after NACT. Identifying the target tumor area using pre-NACT MRI was feasible, and the tumor was safely removed with clear resection margins. | 4 |
| 28 | Lee HS, Kim HJ, Chung IY, et al. Usefulness of 3D-surgical guides in breast conserving surgery after neoadjuvant treatment. Sci Rep. 2021;11(1):3376. | Experimental Tx | 39 | We used 3D printed-breast surgical guides (3DP-BSG) to designate the original tumor area from the pre-treatment magnetic resonance imaging (MRI) during breast-conserving surgery (BCS) in breast cancer patients who received neoadjuvant systemic therapy (NST). | With 3DP-BSG for BCS in breast cancer patients receiving NST, the original tumor area can be identified and marked directly on the breast, which is useful for surgery. | 2 |
| 29 | Santiago L, Adrada BE, Caudle AS, Clemens MW, Black DM, Arribas EM. The role of three‐dimensional printing in the surgical management of breast cancer. Journal of surgical oncology. 2019;120(6):897-902. | Review/Other- Dx and Tx | 1 | A patient-specific 3-dimensional printed model (3DPM) of a woman with breast cancer was created. | After review of the 3D printed model, the patient and surgeon agreed on breast-conserving surgery. Use of patient-specific 3DPM in the setting of breast cancer may aid patient decision making and surgical planning, leading to enhanced surgical and oncological outcomes. | 4 |
| 30 | Jablonka EM, Wu RT, Mittermiller PA, Gifford K, Momeni A. 3-DIEPrinting: 3D-printed models to assist the intramuscular dissection in abdominally based microsurgical breast reconstruction. Plastic and Reconstructive Surgery Global Open. 2019;7(4). | Review/Other-Tx | 1 | Harvest of the deep inferior epigastric vessels for microsurgical breast reconstruction can be complicated by an intricate and lengthy subfascial dissection. Although multiple preoperative imaging modalities exist to help visualize the vascular anatomy and assist in perforator selection, few can help clearly define the intramuscular course of these vessels. | The authors introduce their early experience with 3D-printed anatomical modeling (to-scale) of the infraumbilical course of the deep inferior epigastric subfascial vascular tree to better assist in executing the intramuscular dissection. | 4 |
| 31 | Chae MP, Rozen WM, McMenamin PG, Findlay MW, Spychal RT, Hunter-Smith DJ. Emerging applications of bedside 3D printing in plastic surgery. Frontiers in surgery. 2015;2:25. | Review/Other-Tx | n/a | Existing uses of 3D printing in plastic surgery practice spanning the spectrum from templates for facial transplantation surgery through to the formation of bespoke craniofacial implants to optimize post-operative esthetics are described. | They discuss the potential of 3D printing to become an essential office-based tool in plastic surgery to assist in preoperative planning, developing intraoperative guidance tools, teaching patients and surgical trainees, and producing patient-specific prosthetics in everyday surgical practice. | 4 |
| 32 | Chae MP, Hunter-Smith DJ, Rostek M, Smith JA, Rozen WM. Enhanced preoperative deep inferior epigastric artery perforator flap planning with a 3D-printed perforasome template: technique and case report. Plastic and Reconstructive Surgery Global Open. 2018;6(1). | Review/Other-Tx | 1 | They describe a 3D-printed template that can be used preoperatively to mark out a patient’s individualized perforasome for flap planning in DIEP flap surgery. | They describe a new technique of 3D printing a patient-specific perforasome template that can be used preoperatively to infer perforasomes and aid flap design. | 4 |
| 33 | Chae MP, Hunter-Smith DJ, Spychal RT, Rozen WM. 3D volumetric analysis for planning breast reconstructive surgery. Breast Cancer Res Treat. 2014;146(2):457-460. | Review/Other-Tx | 1 | They describe a novel approach to volumetric analysis of the breast, through the creation of a haptic, tactile model, or 3D print of scan data. | Preoperative planning, including volumetric analysis can be used as a tool to aid esthetic outcomes and attempt to reduce operative times in post-mastectomy breast reconstruction surgery. The combination of accurate volume calculations and the production of 3D-printed haptic models for tactile feedback and operative guidance are evolving techniques in volumetric analysis and preoperative planning in breast reconstruction. | 4 |
| 34 | Hummelink S, Verhulst AC, Maal TJ, Ulrich DJ. Applications and limitations of using patient-specific 3D printed molds in autologous breast reconstruction. European journal of plastic surgery. 2018;41(5):571-576. | Review/Other-Tx | 6 | Share their preliminary experiences with creating patient-specific, three-dimensional (3D) printed breast molds for intraoperative use based on 3D stereophotogrammetry. and its clinical possibilities and limitations in practice. | Patient-specific breast templates are inexpensive and relatively easy to design, while being practical and convenient to obtain insight in the dimensions of the desired breast during reconstruction, according to the operating surgeons. Patient selection is however critical, as patients must have sufficient donor volume and/or satisfying breast shape to be able to use the template to its full potential.Level of evidence: Level IV, therapeutic study. | 4 |
| 35 | DeFazio MV, Arribas EM, Ahmad FI, et al. Application of Three-Dimensional Printed Vascular Modeling as a Perioperative Guide to Perforator Mapping and Pedicle Dissection during Abdominal Flap Harvest for Breast Reconstruction. J Reconstr Microsurg. 2020;36(5):325-338. | Experimental Tx | 50 | Presenting early experience using this tool to navigate deep inferior epigastric artery (DIEA) topography and evaluate its impact on operative efficiency and clinical outcomes. | Overall, complete concordance was observed between 3DVM and operative findings with regards to perforator number, source-vessel origin, and DIEA branching pattern. By comparison, CTA interpretation of these parameters inaccurately identified branching pattern and perforator source-vessel origin in 28 and 33% of hemi-abdomens, respectively (p = 0.045 and p = 0.02). Compared with operative measurements, the average MOE for perforator localization using 3DVM was significantly lower than that obtained from CTA alone (0.81 vs. 8.71 mm, p < 0.0001). Reference of 3D-printed models, intraoperatively, was associated with a mean reduction in flap harvest time by 21 minutes (60.7 vs. 81.7 minutes, p < 0.001). Although not statistically significant, rates of perforator-level injury, microvascular insufficiency, and fat necrosis were lower among patients mapped using 3DVM. | 2 |
| 36 | Mehta S, Byrne N, Karunanithy N, Farhadi J. 3D printing provides unrivalled bespoke teaching tools for autologous free flap breast reconstruction. Journal of Plastic, Reconstructive & Aesthetic Surgery. 2016;69(4):578-580. | Review/Other-Tx | NA | In this study, we applied 3D printing to autologous reconstructive breast surgery to create a patient specific model to help plan and teach DIEP flap breast reconstruction. | This is the first application of 3D printing in breast reconstruction. | 4 |
| 37 | Chae MP, Hunter-Smith DJ, Chung RD, Smith JA, Rozen WM. 3D-printed, patient-specific DIEP flap templates for preoperative planning in breast reconstruction: a prospective case series. Gland Surg. 2021;10(7):2192-2199. | Experimental Tx | 20 | A 3D-printed model provides tactile feedback that facilitates superior understanding. Hence, we have 3D-printed patient-specific deep inferior epigastric artery perforator (DIEP) templates, in an affordable and convenient manner, for preoperative planning. | 3D printing time took mean 18 hours and 123.7 g of plastic filament, which calculates to a mean material cost of AUD 8.25. DIEP templates accurately identified the perforators and reduced intraoperative perforator identification by 7.29 minutes (P=0.02). However, the intramuscular dissection time was not affected (P=0.34). Surgeons found the template useful for preoperative marking (8.6/10) and planning (7.9/10), but not for intramuscular dissection (5.9/10). There were no immediate flap-related complications. | 2 |
| 38 | Chen K, Feng CJ, Ma H, et al. Preoperative breast volume evaluation of one-stage immediate breast reconstruction using three-dimensional surface imaging and a printed mold. J Chin Med Assoc. 2019;82(9):732-739. | Experimental-DX and Tx | 19 | In this study, we compared breast volume estimation using three-dimensional (3D) surface imaging with magnetic resonance imaging (MRI) to determine the accuracy of breast volume measurements. Further, a 3D printing mold for facilitating autologous breast reconstruction intraoperatively is described. | There was a strong linear association between breast volume measured using the two different methods and water displacement of mastectomy specimens when a Pearson correlation was used (3D surface image: r = 0.925, p < 0.001; MRI: r = 0.915, p < 0.001). Bland-Altman plots demonstrated no proportional bias between the assessment methods. The coefficient of variation was 52.7% for 3D surface imaging and 59.9% for MRI. The volume of six breasts was evaluated by both measurements and the intraclass correlation coefficient was 0.689 for 3D surface image (p = 0.043) and 0.743 for MRI (p = 0.028). | 3 |
| 39 | Ogunleye AA, Deptula PL, Inchauste SM, et al. The utility of three-dimensional models in complex microsurgical reconstruction. Arch Plast Surg. 2020;47(5):428-434. | Experimental-Tx | 116 | A retrospective review of patients undergoing reconstructive breast microsurgery procedures from 2017 to 2019 who received computed tomography angiography (CTA) scans only or with 3D models for preoperative surgical planning were performed. | Fifty-eight abdominal-based breast free-flaps performed using conventional CTA were compared with a matched cohort of 58 breast free-flaps performed with 3D model print. There was no flap loss in either group. There was a significant reduction in flap harvest time with use of 3D model (CTA vs. 3D, 117.7±14.2 minutes vs. 109.8±11.6 minutes; P=0.001). In addition, there was no change in preoperative decision on type of flap harvested in all cases in 3D print group (0%), compared with 24.1% change in conventional CTA group. | 2 |
| 40 | Tomita K, Yano K, Taminato M, Nomori M, Hosokawa K. DIEP Flap Breast Reconstruction in Patients with Breast Ptosis: 2-Stage Reconstruction Using 3-Dimensional Surface Imaging and a Printed Mold. Plast Reconstr Surg Glob Open. 2017;5(10):e1511. | Experimental-Tx | 8 | In the present study, the use of three-dimensional (3D) imaging and printing technologies in deep inferior epigastric artery perforator (DIEP) flap breast reconstruction technologies were applied to the reconstruction of breasts with ptosis. | All flaps were engrafted without any major perioperative complications during both the initial and DIEP flap surgeries. Objective assessment of cosmetic outcome revealed that good breast symmetry was achieved in all cases.  The method described here may allow even inexperienced surgeons to achieve reconstruction of symmetrical, non-ptotic breasts with ease and in a short time. While the requirement of two surgeries is a potential disadvantage, our method will be particularly useful in cases involving TEs, i.e., delayed reconstruction or immediate reconstruction involving significant skin resection. | 3 |
| 41 | Poulin E, Gardi L, Fenster A, Pouliot J, Beaulieu L. A novel approach for real-time, personalized breast HDR brachytherapy treatment using 3D printing technology. Brachytherapy. 2014;13:S18. | Review/Other-Tx | n/a | In this work, we present an innovative approach for real-time and personalized 3D ultrasound (3DUS) planning in breast HDR brachytherapy. | We have devised a simple, fast, and efficient method for real-time and personalized 3DUS breast HDR brachytherapy treatment using inverse planning and 3D printing technology. This novel personalized approach could be easily extended to prostate and other sites. | 4 |
| 42 | Aristei C, Lancellotta V, Piergentini M, et al. Individualized 3D-printed templates for high-dose-rate interstitial multicathether brachytherapy in patients with breast cancer. Brachytherapy. 2019;18(1):57-62. | Experimental-Tx | 13 | The present article reports the results of a study that aimed at producing and validating a 3D-printed template to aid in target volume localization for multicatheter interstitial brachytherapy in patients with breast cancer. | Visual assessment and X-ray findings showed the 3D-printed target volume always fell within the standard volume in all 13 patients. The intraclass correlation coefficient indicated moderate agreement for both the medial and the lateral skin projections. | 3 |
| 43 | Robar JL, Moran K, Allan J, et al. Intrapatient study comparing 3D printed bolus versus standard vinyl gel sheet bolus for postmastectomy chest wall radiation therapy. Pract Radiat Oncol. 2018;8(4):221-229. | Experimental TX | 16 | This patient study evaluated the use of 3-dimensional (3D) printed bolus for chest wall radiation therapy compared with standard sheet bolus with regard to accuracy of fit, surface dose measured in vivo, and efficiency of patient setup. By alternating bolus type over the course of therapy, each patient served as her own control. | This study demonstrates 3D printed bolus in postmastectomy radiation therapy improves fit of the bolus and reduces patient setup time marginally compared with standard vinyl gel sheet bolus. The time savings on patient setup must be weighed against the considerable time needed for the 3D printing process. | 2 |
| 44 | Yang K, Park W, Ju SG, et al. Heart-sparing radiotherapy with three-dimensional printing technology after mastectomy for patients with left breast cancer. Breast J. 2019;25(4):682-686. | Experimental Tx | 28 | The purpose of this study was to analyze the effectiveness of electron beam therapy (EBT) with patient-tailored bolus (PTB) using three-dimensional printing technology to reduce heart and lung doses during post-mastectomy radiotherapy (PMRT) | A customized virtual bolus was designed for PMRT to compensate for surface irregularities on computed tomography images and developed optimized plans for EBT. As comparison between the PTB and tangential plans, the PTB plan reduced unnecessary exposure to heart and ipsilateral lung with better target coverage compared with the tangential technique. | 1 |
| 45 | He C, Zhang S, Shi L. Three-Dimensionally-Precise Breast Conformal Device for IMRT in Breast Cancer Patients Treated With Breast-Conserving Surgery-A Pilot Randomized Controlled Trial. Technol Cancer Res Treat. 2020;19:1533033820971563. | Experimental Tx | 30 | To examine the accuracy and efficiency of breast radiotherapy after breast-conserving surgery of a novel 3-dimensional (3D) printing tissue compensator technology, the 3D-precise breast conformer, compared with a usual compensator and an unstructured compensator. | The 3D-BCT showed the best homogeneity index (HI) (0.08 ± 0.03) and conformity index (CI) (0.95 ± 0.03), while the NST group showed the worst HI (0.34 ± 0.07) and CI (0.78 ± 0.06), with the ST group between the 2 (HI: 0.15 ± 0.05; CI: 0.87 ± 0.04) (all P < 0.01). The common tissue compensation membrane could lead to 95-100% of the prescription dose covering 85-95% of the target volume, and the uniformity and conformability of the target dose were improved overall compared with the NST group. In the 3D-BCT group, 100% of the prescription dose covered the target volume of 95-100%. | 2 |
| 46 | Park SY, Choi CH, Park JM, Chun M, Han JH, Kim JI. A Patient-Specific Polylactic Acid Bolus Made by a 3D Printer for Breast Cancer Radiation Therapy. PLoS One. 2016;11(12):e0168063. | Review/Other-Tx | n/a | The aim of this study was to assess the feasibility and advantages of a patient-specific breast bolus made using a 3D printer technique. | There was a good agreement between the dose distribution for a virtual bolus generated by the TPS and PLA bolus. The difference in dose distribution between the virtual bolus and Super-Flex bolus was significant within the bolus and breast due to unwanted air gaps. The average differences between calculated and measured doses in a 200 and 300 cc with PLA bolus were not significant, which were -0.7% and -0.6% for 3mm, and -1.1% and -1.1% for 5 mm, respectively. With the Super-Flex bolus, however, significant dose differences were observed (-5.1% and -3.2% for 3mm, and -6.3% and -4.2% for 5 mm). | 4 |
| 47 | Park K, Park S, Jeon MJ, et al. Clinical application of 3D-printed-step-bolus in post-total-mastectomy electron conformal therapy. Oncotarget. 2017;8(15):25660-25668. | Experimental Tx | 6 | the purpose of this study was to confirm whether 3D-printed boluses are better suited instead of conventional step boluses for use during electron conformal therapy. | Based on in-vivo measurements, it was found that the 3D-printed boluses improved the precision of the dose absorbed by the chest wall to 3%; in contrast, the use of the conventional step bolus resulted in a dose uncertainty of up to 6%. Furthermore, the homogeneity of the dose distribution both on the surface and within the chest wall was improved. In addition, the dose absorbed by the normal organs was reduced by up to 20%. | 3 |
| 48 | Poulin E, Gardi L, Fenster A, Pouliot J, Beaulieu L. Towards real-time 3D ultrasound planning and personalized 3D printing for breast HDR brachytherapy treatment. Radiother Oncol. 2015;114(3):335-8. | Review/Other-Tx | n/a | Two different end-to-end procedures were tested for real-time planning in breast HDR brachytherapy treatment. Both methods are using a 3D ultrasound (3DUS) system and a freehand catheter optimization algorithm. | we have demonstrated a proof-of-concept to perform simple, fast, and efficient real-time planning and personalized 3DUS breast HDR brachytherapy treatment using inverse planning and 3D printing technology. | 4 |
| **Supplementary Table**. Grading of each included study with a strength of evidence assessment according to ACR Appropriateness Criteria Evidence Document.^2^ Studies were categorized as either primarily diagnostic (Dx), therapeutic (Tx), or both (Dx and Tx) along with a designation of observational, experimental, or review/other category. The review/other category is designated for studies that did not meet the definitions the ACR Evidence Document^2^ for observational or experimental studies. | | | | | | |
